# Green nail syndrome following nail art

**DOI:** 10.1093/omcr/omaf300

**Published:** 2026-01-28

**Authors:** Yixuan Zhang, Lu Liu

**Affiliations:** Department of Dermatology, The Fourth Affiliated Hospital of Soochow University (Suzhou Dushu Lake Hospital, Medical Center of Soochow University), No. 9 Chongwen Road, Suzhou 215125, Jiangsu Province, China; Department of Burns and Plastic Surgery, Affiliated Suzhou Hospital of Nanjing Medical University, No. 242 Guangji Road, Suzhou 215002, Jiangsu Province, China

**Keywords:** green nail syndrome, nail art, topical fluoroquinolone therapy

**Figure 1:**
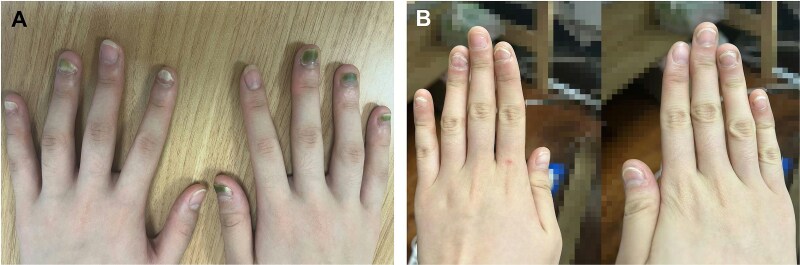
(A) Green discoloration and onycholysis of multiple fingernails after a nail art procedure. (B) Clinical improvement following topical levofloxacin treatment.

A healthy 17-year-old girl developed green discoloration and partial onycholysis of multiple fingernails shortly after a single gel nail art procedure (Fig. 1A). She reported no pain, swelling, or systemic symptoms, and denied frequent water exposure, prior nail disease, or medication use. Examination revealed green discoloration and distal separation of several nails without periungual inflammation. Both fungal and bacterial cultures of the nail clippings were negative. Based on the characteristic appearance and temporal relationship with cosmetic manipulation, green nail syndrome (GNS) was diagnosed. She was treated with topical levofloxacin 0.5% ophthalmic solution applied twice daily by instilling drops under the distal nail edge, resulting in marked improvement and nail reattachment within 10 days (Fig. 1B). This rapid ‘reattachment’ reflected resolution of infection and restoration of nail bed adhesion rather than true nail regrowth.

GNS is most commonly caused by *Pseudomonas aeruginosa* and typically involves a single nail, while multiple nail involvement is unusual. Other pathogens like *Citrobacter species, Klebsiella species*, and *Staphylococcus aureus* could also be involved [1, 2]. Several risk factors have been identified, including repeated nail trauma [1], chronic exposure to moist environments, and occlusive conditions [3]. Pre-existing nail disorders like onychomycosis or onycholysis may also create spaces that favor bacterial growth [4]. In this case, the nail art procedure likely caused microtrauma that facilitated colonization, highlighting the importance of proper sterilization of nail art tools to reduce the risk of GNS. Although culture confirmation is desirable, negative results are frequently reported in GNS [5], as sampling limitations and false negatives are common. In such cases, diagnosis relies heavily on recognizing the characteristic green discoloration and clinical context. Topical antibiotics, especially fluoroquinolones, are effective and well tolerated, offering a safe alternative to systemic therapy in adolescents [2]. This case emphasizes the importance of recognizing characteristic findings, understanding potential triggers, and initiating early topical therapy to prevent chronic nail changes.
